# New Sequence Type ST3449 in Multidrug-Resistant *Pseudomonas aeruginosa* Isolates from a Cystic Fibrosis Patient

**DOI:** 10.3390/antibiotics10050491

**Published:** 2021-04-23

**Authors:** Catalina Díaz-Ríos, Marta Hernández, David Abad, Laura Álvarez-Montes, Athanasia Varsaki, David Iturbe, Jorge Calvo, Alain A. Ocampo-Sosa

**Affiliations:** 1 Instituto de Investigación Sanitaria Marqués de Valdecilla (IDIVAL), 39011 Santander, Spain; catalina_diaz91@hotmail.com (C.D.-R.); lalvarez@idival.org (L.Á.-M.); 2 Instituto Tecnológico Agrario de Castilla y León (ITACyL), 47071 Valladolid, Spain; hernandez.marta@gmail.com (M.H.); AbaGarDa@itacyl.es (D.A.); 3 Centro de Investigación y Formación Agraria (CIFA), 39600 Muriedas, Spain; avarsaki@gmail.com; 4 Servicio de Neumología, Hospital Universitario Marqués de Valdecilla, 39008 Santander, Spain; david.iturbe@scsalud.es; 5 Servicio de Microbiología, Hospital Universitario Marqués de Valdecilla, 39008 Santander, Spain; jorge.calvo@scsalud.es

**Keywords:** *Pseudomonas aeruginosa*, sequence type ST3449, multi-drug resistance, cystic fibrosis, biofilm, virulence

## Abstract

*Pseudomonas aeruginosa* is one of the most critical bacterial pathogens associated with chronic infections in cystic fibrosis patients. Here we show the phenotypic and genotypic characterization of five consecutive multidrug-resistant isolates of *P. aeruginosa* collected during a month from a CF patient with end-stage lung disease and fatal outcome. The isolates exhibited distinct colony morphologies and pigmentation and differences in their capacity to produce biofilm and virulence potential evaluated in larvae of *Galleria mellonella*. Whole genome-sequencing showed that isolates belonged to a novel sequence type ST3449 and serotype O6. Analysis of their resistome demonstrated the presence of genes *bla*_OXA-396_, *bla*_PAO_, *aph(3’)-IIb*, *catB*, *crpP* and *fosA* and new mutations in chromosomal genes conferring resistance to different antipseudomonal antibiotics. Genes *exoS*, *exoT*, *exoY*, *toxA*, *lasI*, *rhlI* and *tse1* were among the 220 virulence genes detected. The different phenotypic and genotypic features found reveal the adaptation of clone ST3449 to the CF lung environment by a number of mutations affecting genes related with biofilm formation, quorum sensing and antimicrobial resistance. Most of these mutations are commonly found in CF isolates, which may give us important clues for future development of new drug targets to combat *P. aeruginosa* chronic infections.

## 1. Introduction

*Pseudomonas aeruginosa* is among the most dreaded opportunistic human pathogens that affect the airways of cystic fibrosis (CF) individuals. This bacterium is notorious for its metabolic versatility and innate resistance to many antibiotics due to the permeability barrier conferred by its Gram-negative outer membrane. *P. aeruginosa* also has a tendency to form specialized bacterial communities called biofilms, which make bacterial cells impenetrable to therapeutic concentrations of antibiotics [1].

Respiratory tract infection in CF patient usually initiates with persistent infections by *P. aeruginosa* that are frequently eradicated by antibiotic treatments. Recolonization may take place, either by the same recalcitrant strain or other different strains, which can establish permanently in the airways leading to chronic infections in the respiratory tract of CF patients. Long-term colonization of the CF lung by *P. aeruginosa* generally occurs by single lineages, which are clonal to the strain acquired during initial colonization and can persist in the airway of a patient most of their lives [2]. *P. aeruginosa* displays high virulence at initial stages of infection and it is relatively susceptible to antibiotics, but during the transition to a chronic way of survival it undergoes several adaptations to overcome the hostile environment of the lung. Among the characteristics *P. aeruginosa* exhibits at chronic phases of infection the presence of a mucoid phenotype, reduced expression of virulence factors, reduced motility, enhanced biofilm formation ability, high mutation rates, appearance of small colony variants (SCV), pigmentation changes and resistance to antimicrobial agents [3,4].

Development of multidrug resistance (MDR) in *P. aeruginosa* during persistent infections is induced either by the accumulation of pathoadaptive chromosomal mutations or the acquisition of antimicrobial resistance determinants by horizontal gene transfer [2]. Increased ratios of resistance to beta-lactams, aminoglycosides and fluoroquinolones are usually detected among CF isolates. Moreover, some estimates point out that up to 40% of isolates from CF patients are MDR [5,6].

A deeper analysis in order to understand the role of mutations in the adaptive process of *P. aeruginosa* during CF, in particular of those related with the development of resistance to antimicrobial agents, is crucial for future development of new drug targets and the implementation of more efficient antibiotic treatments. In that sense, the advent of high-throughput sequencing technologies in combination with bioinformatics analysis has provided broad insights into bacterial evolution and pathogenesis, including polymorphisms associated with increased antimicrobial resistance and adaptation to specific niches. Some recent studies have employed whole genome sequencing (WGS) to examine the genetic variations of *P. aeruginosa* population during long-term infection of CF individuals [7,8,9,10,11,12].

With this work we aim to characterize five consecutive multidrug-resistant *P. aeruginosa* isolates belonging to a novel sequence type (ST3449) collected from a CF patient with end-stage pulmonary function. This study is a descriptive analysis where isolates were whole-genome sequenced by means of the Illumina Miseq platform to identify the antimicrobial resistance and virulence genes inventory. Our data provide new information about mutations in genes conferring antimicrobial resistance and in virulence factors that will need further investigation for a better understanding about the evolution of this clone.

## 2. Results and Discussion

### 2.1. Isolation, Phenotypic Features and Antimicrobial Susceptibility of the CF P. aeruginosa Strains

*P. aeruginosa* isolates MS6000, MS6002, MS6003, MS6004 and MS6005 were sequentially collected during a month (three first isolates from sputum, and the latter two from tracheal aspirate) from a CF patient with end-stage pulmonary disease who died before lung transplantation. Isolate numbering was assigned according to the consecutive date of isolation, that is to say MS6000 was the first strain isolated and MS6005 the last one. Isolates were identified as *P. aeruginosa* by means of the MALDI-TOF/Vitek MS instrument. The isolates displayed different colony morphology and pigmentation and all of them produced hemolysis (Table 1).

All the isolates were non-susceptible to aztreonam, cefepime, ceftazidime, piperacillin, piperacillin/tazobactam, imipenem, ciprofloxacin and levofloxacin. Conversely, all of them were susceptible to ceftazidime/avibactam and ceftolozane/tazobactam combinations. Except MS6002 that was resistant to meropenem and intermediate to doripenem, the rest of isolates were susceptible to these agents. All isolates were non-susceptible to gentamicin with the exception of MS6004. In the case of tobramycin, isolates MS6000, MS6003 and MS6005 were resistant to this drug. Amikacin was the most effective aminoglycoside antibiotic, as only isolate MS6003 showed resistance to this agent. Isolates MS6002 and MS6003 display resistance to colistin, but the rest remained susceptible. In the case of fosfomycin only MS6003 and MS6004 retained its susceptibility to this antibiotic. All isolates were categorized as XDR, except MS6004 that was considered as MDR (Table 2).

### 2.2. Genome Analysis of the CF P. aeruginosa Isolates

Whole genome sequencing (WGS) of the five CF *P. aeruginosa* isolates was conducted in order to examine the molecular basis of their antimicrobial resistance and clonal relatedness, and their virulence genes repertoire. Summary and statistics for the genome sequencing of the *P. aeruginosa* isolates using the in-house bioinformatics pipeline TORMES version 1.0 [13] are detailed in Table 3.

**Figure 1:**
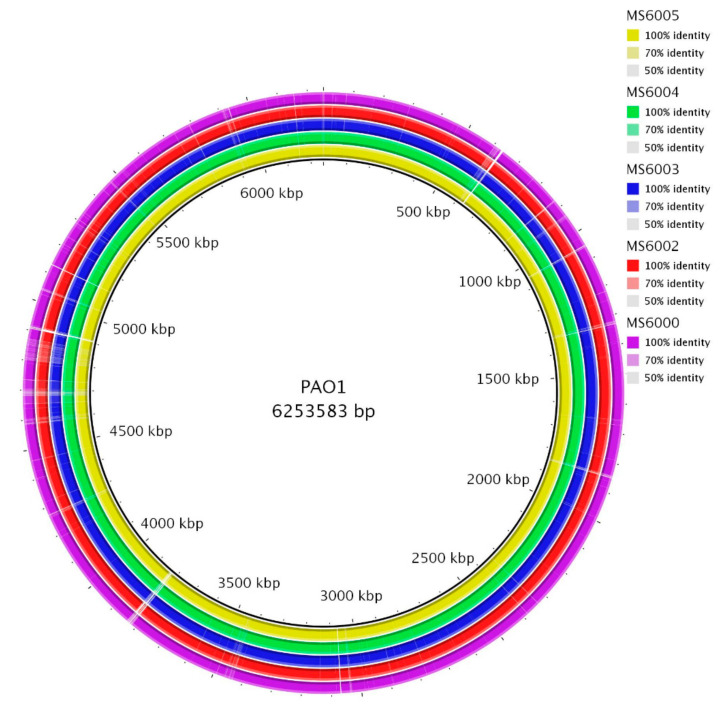
Circular representation of the CF *P. aeruginosa* isolates genomes. Comparison was performed using the blast ring image generator (BRIG) software [14]. Each genome is represented by a ring with different colors shown in the legend at the upper right panels. The genome of *P. aeruginosa* PAO1 (GenBank: JIEO00000000.1) was used as a reference sequence, represented in the innermost ring. The second innermost ring shows MS6005 genome and followed by isolates MS6004, MS6003, MS6002 and MS6000.

Genomic differences between CF isolates were determined by aligning the genomes using BRIG software [14] (Figure 1). Alignment of the assembled contig sequences of draft genomes of CF isolates with PAO1 showed a high level of conservation along their chromosomes. Based on the average genome length of the five CF isolates (6,355,286 bp) and the size of the PAO1 genome (6,253,583 bp, GenBank: JIEO00000000.1) used as a reference, we estimated that the average percentage of genome covered for the 5 CF isolates was 98.4%. MS6000 has an estimated genome size of 6,326,892 bp (5815 ORFs, GC% of 66.37), MS6002 of 6,324,360 bp (5830 ORFs, GC% of 66.38), MS6003 of 6,339,440 bp (5836 ORFs, GC% of 66.35), MS6004 of 6,451,152 bp (5953 ORFs, GC% of 66.37) and MS6005 of 6,334,587 bp (5826 ORFs, GC% of 66.36) (Table 3). The pangenome consisted of 5994 genes, with a core genome of 5753 (96%) genes and a shell genome of 241 (4%) genes. Core genome represents a pool of conserved genes, which are present in all genomes included in the analysis. The shell genes are those moderately common in the pangenome, including genes that are flexibly gained and lost, reflecting the evolution of a lineage and adaptation process of an organism to a particular niche. Soft-core category represents those genes present in 95% of genomes analyzed and cloud cluster is formed by genes present in less than 15% of genomes analyzed. In our analysis these two latter categories of genes were not represented. No plasmids were detected in any of the isolates.

The MLST analysis showed that all CF *P. aeruginosa* isolates represented a novel sequence type ST3449 with the following housekeeping gene allele combination: *acsA* (28), *aroE* (150), *guaA* (11), *mutL* (7), *nuoD* (27), *ppsA* (6) and *trpE* (7). To the best of our knowledge this is the first report of ST3449 in isolates of *P. aeruginosa* from a CF patient.

The *P. aeruginosa* serotyper (PAst) program [15] was employed to perform the in silico serotyping of the isolates based on the sequences of the O-specific antigen (OSA) gene cluster. Typeability of *P. aeruginosa* is often lost in those isolates recovered from CF patients due to the adaptive process they undergo during chronic infections. The CF isolates analyzed here belonged to serotype O6. Earlier studies have also shown the presence of serotype O6 among CF *P. aeruginosa* isolates [16,17], which is often associated with poor clinical outcomes [6]. A recent Spanish national survey demonstrated that O6 was among the most prevalent serotypes in the population studied [18].

#### 2.2.1. Resistome Analysis

Analysis of the horizontally acquired genes using the ResFinder database [19] revealed that all the isolates harbored resistance genes to beta-lactams (*bla*_OXA-396_ and *bla*_PAO_), aminoglycosides (*aph(3’)-IIb*), fluoroquinolones (*crpP*), phenicols (*catB7*) and fosfomycin (*fosA*). The presence of missense and nonsense mutations in genes known to confer resistance to the main groups of antipseudomonal agents such as beta-lactams, aminoglycosides, fluoroquinolones, polymixins and fosfomycin is shown in Table 4. A comprehensive list including all the mutations predicted by the breseq program [20] in the whole-genome sequences of the CF isolates analyzed in this work is available in the Supplementary Table S1.

Resistance to beta-lactams in *P. aeruginosa* is primarily due to a contribution of several mechanisms such as overexpression of intrinsic beta-lactamase AmpC, horizontally acquired beta-lactamases carried by conjugative plasmids, low outer membrane permeability (porin mediated) and/or overproduction of RND (resistance-nodulation-division) efflux pumps, mainly MexAB-OprM and MexXY-OprM [21]. Role of penicillin binding proteins (PBPs) in beta-lactams resistance has also been demonstrated [22].

As mentioned before, the resistome analysis revealed that the five isolates harbored the beta-lactamase genes *bla*_OXA-396_ and *bla*_PAO_. Previous studies demonstrated the high prevalence of these genes in *P. aeruginosa* isolates from different sources and their contribution to antimicrobial resistance in this species [23,24,25,26,27]. According to the ResFinder tool on WGS from the five CF isolates, no carbapenemase genes were detected.

A missense mutation in the *ampC* gene leading to a T105A substitution in the beta-lactamase AmpC was found in all the isolates. It was formerly suggested that this mutation was related with reduced susceptibility to oxyiminocephalosporins and imipenem [28], but it did not affect ceftazidime [29]. Other studies have shown this is a common polymorphism in bela-lactam-resistant *P. aeruginosa* isolates [30,31,32,33,34]. However, its relationship with beta-lactam resistance is rather uncertain [31]. Overexpression of *ampC* is generally due to selected mutations in its regulator genes *ampR*, *ampD* and *dacB*. All our isolates presented a stop codon at nucleotide 874 in *ampR* due to the insertion of a thymine at position 860. Polymorphisms G148A, D183Y in AmpD were also detected, which were previously demonstrated not to be involved in *ampC* overexpression [30]. Isolate MS6003 showed an alteration in *ampD* consisting in the addition of a guanine at position 443 of the gene, resulting in a frameshift mutation (Table 4). AmpD homologues, AmpDh2 and AmpDh3 were previously described as negative regulators of AmpC [35]. No alterations were detected in AmpDh2, while mutation A219T in AmpDh3 was identified in all the isolates. This amino acid change in AmpDh3 was described before in multidrug-resistant *P. aeruginosa* isolates from ST175 and ST664 [36,37]. No mutations in the AmpC regulator *dacB* (PBP4) were found in our isolates.

A frameshift mutation was found in MS6000, MS60002 and MS6005 in the *mpl* gene, coding for a UDP-muramic acid/peptideligase, due to a deletion of 14 nucleotides. As far as we know, this deletion in *mpl* is described here for the first time. Inactivation of *mpl* has been shown to be responsible for increased activity of AmpC in *P. aeruginosa* [38,39]. Isolate MS6004 showed a missense mutation G133D in Mpl, previously observed in a CF isolate resistant to beta-lactams [33], but its implication in AmpC activation is unknown. Mutations in *mpl* are frequently found in *P. aeruginosa* isolates from CF individuals as a consequence of long-term colonization [40,41,42].

Mutations in some PBPs were documented in this study. All the isolates showed a missense mutation D329G in PonA (PBP1a) and the addition of the triplet CCG at position 1845 of *ponA* and the amino acid substitution L353Q in MrcB (PBP1b). PBP3a (*pbpC*) was absent in all except one, MS6004 that presented the mutation A104P. The substitution in residue S250N was found in PBP7 (*pbpG*) in all the isolates. Most of these mutations in PBP genes have been previously described, but their contribution to beta-lactams resistance remains to be demonstrated [33,36,43]. The rest of PBPs did not show any alteration.

The multidrug resistance efflux system MexAB-OprM is responsible for the extrusion of several antibiotics, among them, beta-lactams. Mutations accumulated in transcriptional regulators *mexR*, *nalC* and *nalD* lead to overexpression of this efflux pump. Of these three regulators, only NalC was found to display non-synonymous mutations G71E and S209R in all the isolates. These mutations seem to be frequent in multidrug resistant *P. aeruginosa* isolates, however their role in inducing overexpression of MexAB-OprM is unclear [33,36,44,45]. Two missense mutation not previously described (L627R and L684P) were detected in MexB (Table 4). However, we do not know their effect on the expression of this efflux system.

Porin OprD is a primary route of entry of imipenem in *P. aeruginosa*. OprD deficiency or loss from the outer membranes confers certain level of resistance to carbapenems [46]. Several polymorphisms were found in OprD (Table 4), affecting loops 1, 5, 6 and 7 of the porin. Some of these alterations in OprD had been described before with no contribution to carbapenem resistance [45,47,48,49]. Moreover, shortening of the loop 7 by deletion of residues G378 and Y379, here detected, has been associated with increased susceptibility to meropenem [50]. Isolate MS6002 presented a premature stop codon in *oprD* at position 18, thus generating a nonfunctional protein. This non-sense mutation possibly correlates with the resistance phenotype to imipenem displayed by MS6002 with the highest MIC value of this antibiotic among the isolates (Table 2).

Overexpression of MexXY-OprM is one of the major mechanisms of aminoglycoside resistance in *P. aeruginosa*, though this multidrug efflux system has a broad substrate specificity that also comprises quinolones, macrolides and some beta-lactams [51]. Overexpression of this pump is mainly due to mutations in its negative repressor MexZ and the activator AmgS. However, no mutations were detected in our isolates in any of these regulator genes. On the other hand, all the isolates presented the amino acid substitutions W358R, L331V and K329Q in MexX and T543A in MexY. All these mutations in MexXY have been related with resistance to beta-lactams, aminoglycosides and quinolones in a previous study conducted on CF *P. aeruginosa* isolates from the CC274 [33]. Nevertheless, an earlier study showed that these polymorphisms had no implication in resistance to aminoglycosides, as they were also found in strains susceptible to these agents [52]. MS6000, MS6002 and MS6005 presented a new amino acid substitution, A992T in MexY, which contribution to antimicrobial resistance needs to be investigated. Of note, all isolates displayed the amino acid substitutions I181V and E277K in Fmt, a methionyl-tRNA formyltransferase, and T189A in the methylene-tetrahydrofolate dehydrogenase, FolD. These two proteins were formerly determined to be involved in the regulation of MexXY expression [53]. The non-synonymous mutations mentioned above have been described before in CF *P. aeruginosa* isolates [33].

All the isolates displayed two missense mutations, S176A and G695A in FusA2. A previous study demonstrated that mutations in the elongation factor G encoding genes, *fusA1* and *fusA2*, were responsible for high level of aminoglycoside resistance in CF isolates [33]. Mutation T484A was observed in NuoG in all the isolates. This mutation was previously documented in CF *P. aeruginosa* isolates [33]. The *nuoG* gene codes for a type I NADH dehydrogenase, whose inactivation leads to a decrease in aminoglycosides uptake [54].

High aminoglycoside resistance in *P. aeruginosa* is conferred by the presence of acquired aminoglycoside-modifying enzymes (AMEs). As mentioned above in the text, the aminoglycoside phosphotransferase gene, *aph(3′)-IIb* was found in the genomes of all the isolates. This enzyme confers resistance to kanamycin and neomycin but not to gentamicin, tobramycin or amikacin. Therefore, its contribution to the aminoglycoside resistant phenotype of the isolates is not relevant, suggesting that involvement of efflux activity and other mechanisms mentioned above should be playing a role in their aminoglycoside resistance phenotype.

Resistance to fluoroquinolones in *P. aeruginosa* involves a number of mechanisms. Fluroquinolones are substrates of the RND efflux pumps MexAB-OprM, MexXY-OprM, MexCD-OprJ and MexEF-OprN. However, some data suggested that the contribution of the overexpression of these efflux systems to high-level resistance to fluoroquinolones is rather limited [33]. Alterations in the MexAB-OprM and MexXY-OprM efflux systems and their respective regulator have been discussed above in the text. Some missense mutations were observed in all the structural components of MexCD-OprJ (Table 4). These mutations were previously described in multidrug-resistance *P. aeruginosa* isolates from different sources [33,55]. No sequence alterations were detected in the MexCD-OprJ repressor NfxB in any of the isolates, which lead us to think that MexCD-OprJ could be expressing in our isolates.

The positive regulator of the MexEF-OprN, MexT presented a missense mutation F172I and a deletion of eight nucleotides in *mexT* (Table 4), compared to the *mexT* of PAO1. The *mexT* gene missing the 8-bp insert have been described in prior studies and is related with the switch to an active MexT variant [47,56,57,58]. These mutations have been found before in isolates showing either basal or increased activity of MexEF-OprN [47,59]. All the isolates presented the amino acid change D249N in MexS, the negative regulator of MexEF-OprN. According to an earlier study the N249 is considered the wild type functional variant of MexS, present in strains PA14 and PAK [60]. Therefore, our isolates harbor an active MexS variant. It is known that MexS is a negative regulator of MexT. Despite not conducting any gene expression experiment to detect the activity of MexEF-OprN, we could think that as our isolates present a functional MexS, it would be expected that MexT were repressed and consequently activity of MexEF-OprN suppressed. However, overexpression of MexE have been demonstrated before in *P. aeruginosa* isolates with active variants of MexS and MexT, suggesting the existence of other regulatory mechanisms that may affect the expression of MexEF-OprN [45]. Search for mutations in additional regulators of MexEF-OprN such as MvaT and the two-component system (TCS) ParR/S were also carried out. Our isolates did not show any mutations in the *mvaT* gene. Conversely, several alterations were observed in ParR and ParS (Table 4). A premature stop codon (TGA) at position 459 in the *parS* gene found in isolate MS6003, leading to a non-functional two-component sensor ParS. A recent work proposed that mutations in the ParR/S lead to a reduction of *mexS* transcription, thus activating the expression of MexEF-OprN [61].

Resistance to fluoroquinolones also arises as a result of mutations in the quinolone resistance-determining regions (QRDRs) of *gyrA*, *gyrB*, *parC* and *parE*. The typical non-synonymous mutations T83I and D87N were found in GyrA in all the isolates. These mutations are clinically relevant and are often detected in fluoroquinolone-resistant *P. aeruginosa* [33,36,62]. Amino acid substitution S681L in GyrB was found in three of the isolates (Table 4). Missense mutations were also detected in the topoisomerase IV subunits ParC (E91K and V646L) and ParE (G285S) (Table 4). To our knowledge, mutations S681L, V646L and G285S accumulated in the topoisomerases II and IV, respectively, have not been described before. Additional analysis should be carried out to demonstrate whether these mutations are implicated in quinolone resistance or are just randomly selected during chronic infection in the lung of the CF patient. Interestingly, MS6003 was the isolate that accumulated the greatest number of mutations associated to fluoroquinolone resistance, which may correlate with its elevated ciprofloxacin and levofloxacin resistant profiles compared to the rest of isolates (Table 2).

The acquisition of the *crpP* gene, coding for a ciprofloxacin-modifying enzyme [63] was detected in our strains, all of them bearing the non-synonymous mutations K4R and G7D in CrpP, when compared with the CrpP (GenBank accession: NG_062203). A very recent study conducted on a large number of *P. aeruginosa* isolates from Portugal and Spain showed that CrpP proteins with the amino acid substitutions K4R and G7D were the most predominant variants among the population analyzed [64]. A reduced activity of CrpP on ciprofloxacin was previously observed when missense mutations occurred in the amino acid position G7, indicating that this is an essential residue for the enzymatic activity of CrpP [65]. Consequently, the presence of this CrpP variant in our isolates may not contribute to the ciprofloxacin resistance phenotype. On the other hand, based on our information, the implication of the missense mutation K4R on the CrpP activity is yet unknown.

Polymyxin resistance in *P. aeruginosa* is mostly attributable to mutations in the TCSs PhoP/Q, PmrA/B, ParR/S, ColR/S and CprR/S [66]. Several mutations in *pmrA*/*pmrB* and *phoP*/*phoQ* can induce upregulation of the *arnBCADTEF*-*ugd* (*arn*) operon, resulting in modifications of the lipopolysaccharide (LPS) that reduce the interaction of polymyxins with fatty acids and phosphates groups of the LPS [67,68]. When examining the amino acid sequences these TCSs in our isolates we only detected non-synonymous mutations in PmrA (L71R), PmrB (Y345H, V386M), ParR (L153R, S170N) and ParS (H398R) (Table 4). A thorough search in the literature demonstrated that these mutations were described before by other authors in colistin-susceptible strains [69,70], except for the amino acid substitution V386M observed in PmrB in all the isolates and the W153* in ParS found in isolate MS6003 (Table 4). Mutations in ArnB (V302A, E376D) and in other components of the *arn* operon were detected in all the isolates (Table 4 and Table S1), nonetheless the implication of this mutations on the *arn* operon activity and resistance to polymyxins needs to be demonstrated.

With regards to fosfomycin resistance, we carried out the sequence analysis of *murA*, *glpT*, *oprO* and *oprP*, involved in resistance to this agent. The entry of fosfomycin into the bacterium is still not very well understood. Three proteins are known so far to facilitate the entry of fosfomycin in *P. aeruginosa*, the glycerol-3-phosphate transporter, GlpT and porins OprO and OprP. Once in the periplasmic space, fosfomycin inhibits MurA, which initiates peptidoglycan biosynthesis. We did not observe any alteration in *murA*, *glpT* and *oprO*. However, all the isolates possessed the amino acid substitution A98V in OprP. This mutation was reported before, though its relation with fosfomycin resistance is yet unknown [43]. Resistance to fosfomycin is also conferred by FosA, a glutathione S-transferase that modifies enzymatically the antibiotic rendering it ineffective. As previously mentioned, all our isolates harbored the gene *fosA*, whose activity may have been the main contributor to fosfomycin resistance phenotypes showed by some of the isolates.

Altogether, the mutations found in antimicrobial resistance-related genes in this whole-genome sequence analysis may contribute to the antibiotic resistance displayed by our CF *P. aeruginosa* isolates, even when some phenotypic resistance could not be demonstrated in vitro. Therefore, additional in vivo analysis should be carried out to determine the actual role of these mutations developed during the course of chronic pulmonary diseases in CF patients.

#### 2.2.2. Detection of Virulence Related Genes in the CF *P. aeruginosa* Isolates

A total of 220 virulence genes were found among our isolates by comparing the WGS data with the virulence factor database (VFDB), using as reference the *P. aeruginosa* strain PAO1. Supplementary Table S2 contains a list of the virulence factors found in each isolate, according to the VFDB. One of the most represented groups of virulence genes identified were those belonging to the Type III Secretion System (T3SS) machinery (17.7% of the genes), including its secreted exotoxin-coding genes *exoS*, *exoT* and *exoY*. Generally, isolates expressing *exoS* and *exoT* demonstrate an invasive phenotype while those isolates containing *exoU*, have a cytotoxic nature. Our strains did not contain the *exoU* gene. Generally, ExoS and ExoU are mutually exclusive, although some studies have reported the occurrence of isolates harboring both exotoxins [71,72,73,74]. Genes related with flagellar protein biosynthesis were also among the most represented virulence determinants (17%). Genes coding for Type IV pili related functions and twitching motility and those responsible for alginate biosynthesis and regulation represented the 14.5% and 12% of virulence genes, respectively. Several new polymorphisms were found in some genes involved in alginate production such as *algC*, *algE*, algG, *algI*, *algX*, *mucD* and *mucK* (Table S1). Of note, isolate MS6000 presented a new inactivating mutation in *mucA*, due to a substitution of five nucleotides at position 246 (Table S1), generating an anomalous MucA protein. Mutations in *mucA* are related with the transition from the nonmucoid to mucoid condition in *P. aeruginosa* [75,76], which correlates with the mucoid phenotype observed in isolate MS6000 (Table 1). Type VI secretion system (T6SS) genes from Hcp secretion island-1 (H1-T6SS) were also in all five genomes, among them the effector genes *tse1*, *tse2* and *tse3*. The gene *toxA*, coding for exotoxin A, one of the most potent virulence factors produced by *P. aeruginosa*, was observed in all the isolates. Other virulence genes found included those related with biosynthesis of phenazines, pyoverdine and pyochelin, Type II Secretion System, LPS biosynthesis and quorum sensing (QS) genes *rhlI*, *rhlC*, *lasS*, *lasB* and *lasI* (Table S2). All the isolates presented the amino acid substitution S62G and D83E in RhlI (Table S1). These mutations have been described in *P. aeruginosa* isolates from different origins and do not seem to affect the protein function [77]. A frameshift mutation in the master QS signal receptor gene *lasR* due to a deletion of one nucleotide at position 605, was detected in all the isolates. It is known that LasR controls the expression of several genes, including *lasI*, *rhlI* and *rhlR* in laboratory adapted strains, such as PAO1. However, inactivating mutations in *lasR* are commonly found in *P. aeruginosa* isolates from chronic lung infections of CF individuals with an active RhlI/R QS system. This indicates that alternative pathways controlling the expression of RhlI/R system exist and that the *las* system is not entirely essential for virulence of *P. aeruginosa*, as was demonstrated before [77,78].

#### 2.2.3. *Analysis of Genes Related with* *P. aeruginosa* *Hypermutability*

The hypermutator phenotype in *P. aeruginosa* appears as a result of mutations in genes related with in DNA repair processes, especially *mutS*, *mutL* and *uvrD* [33]. In this study we found a number of mutations in *uvrD* and *mutL* but not in *mutS* (Table S1). According to former studies, mutations in *mutL* seem to be the most frequent cause of hypermutability [79]. Mutations in other genes, including those involved in recombination functions (*recB*, *recC*, *recD*, *recJ*, *recN*, *recR*, *recX*, *rdgC*, *rarA*, *uvrB* and *uvrC*) and stress response-related *polA* and *polB* were also detected in our isolates (Table S1). The hypermutator phenotype is commonly found in *P. aeruginosa* isolates from CF patients and it is associated with reduced pulmonary function [80]. Hypermutation in specific genome regions is considered a beneficial mechanism in terms of bacterial fitness and adaptation of *P. aeruginosa* to persist in the CF lung environment. The hypermutator phenotype is usually linked to development of high levels of resistance to antibiotics in *P. aeruginosa* isolates from CF patients [42].

### 2.3. Biofilm Formation Capacities of CF P. aeruginosa Isolates

**Figure 2:**
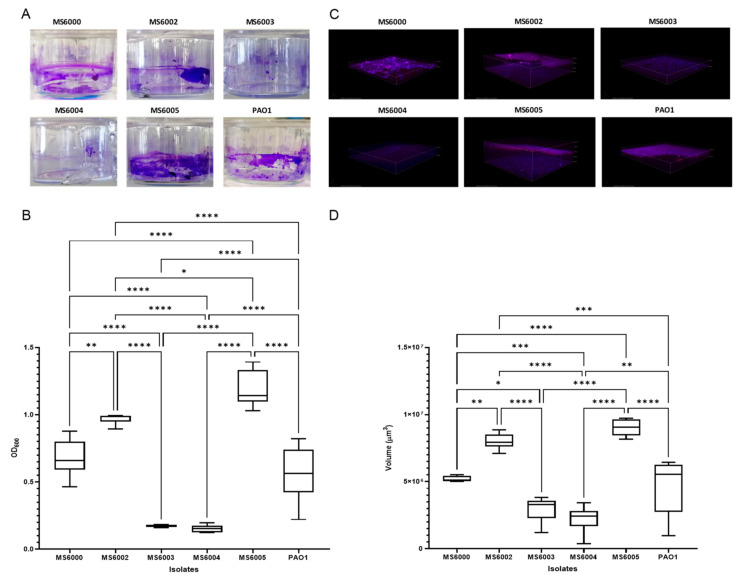
Biofilm formation of the CF *P. aeruginosa* isolates of ST3449. (**A**) Biofilm were developed in 24-well plastic plates and quantified after crystal violet-staining. (**C**) Representative three-dimensional (3D) reconstructions of confocal laser scanning microscopy (CLSM) of *P. aeruginosa* biofilm developed on coated 4-well chamber slides and stained with SYPRO Ruby and DAPI. Representative extracted z images and their respective xy and xz planes are shown. (**B**,**D**) Corresponding levels of biofilm production represent mean values and standard deviations obtained from six independent experiments. Dunnett’s multiple-comparison test was performed and *, **, *** and **** refer to *p* < 0.05, *p* < 0.01, *p* < 0.001 and *p* < 0.0001 significant difference, respectively.

The ability of the CF *P. aeruginosa* isolates to form biofilm was visualized and quantified by using both the crystal violet (CV) and the combination of fluorescent dye SYPRO Ruby/DAPI (Figure 2). All the isolates grew well and were able to produce biofilm. According to the CV biofilm quantification results isolates MS6003 (O.D = 0.17 ± 0.008) and MS6004 (O.D = 0.15 ± 0.02) were classified as moderate biofilm producers, while isolates MS6000 (O.D = 0.68 ± 0.14), MS6002 (O.D = 0.97 ± 0.04) and MS6005 (O.D = 1.19 ± 0.13) were considered as strong biofilm producers. Biofilm production by the CV staining was compared among the isolates and *P. aeruginosa* PAO1 was used as a reference strain (O.D = 0.56 ± 0.21) (Figure 2B). We used confocal laser scanning microscopy (CSLM) to visualize the five CF *P. aeruginosa* isolates biofilm production and architecture (Figure 2C). SYPRO Ruby/DAPI fluorescent staining illustrated significant differences in total biofilm matrix quantity among isolates (Figure 2C). Isolates MS6005 (9.03 ± 0.61 × 10^6^ µm^3^) and MS6002 (7.99 ± 0.59 × 10^6^ µm^3^) displayed the largest volume of biofilm matrix, followed by isolate MS6000 (5.20 ± 0.21 × 10^6^ µm^3^), which showed similar values to PAO1 (4.68 ± 2.12 × 10^6^ µm^3^), while isolates MS6003 (2.96 ± 0.94 × 10^6^ µm^3^) and MS6004 (2.24 ± 1.01 × 10^6^ µm^3^) showed less matrix substance production (Figure 2D). There was a substantial correlation between the two quantification methods (R^2^ = 0.782) (Figure S1).

Biofilm production is of particularly concern because of its association with resistance to antimicrobial agents and evasion to host immune response mechanisms. When *P. aeruginosa* adopts the biofilm form it can easily adapt to the severe inflammatory conditions and antibiotic selective pressure during long periods without eradication [81].

Exopolysaccharides are known to play an important role in the biofilm formation and maintenance. Most of *P. aeruginosa* strains, including PAO1, rely on Pel and Psl polysaccharides to form biofilm and interact with host cells and there is a complex gene network involved in regulation and production of biofilm [82].

Biofilm production is of particular concern because of its association with resistance to antimicrobial agents and evasion to host immune response mechanisms. When *P. aeruginosa* adopts the biofilm form it can easily adapt to the severe inflammatory conditions and antibiotic selective pressure during long periods without eradication [81].

Exopolysaccharides are known to play an important role in the biofilm formation and maintenance. Most of *P. aeruginosa* strains, including PAO1, rely on Pel and Psl polysaccharides to form biofilm and interact with host cells and there is a complex gene network involved in regulation and production of this structure [82].

Similar to other studies, we observed that our CF isolates were able to produce different levels of biofilm in vitro. The WGS analysis revealed several polymorphisms and mutations in genes related with production the aforementioned exopolysaccharides and other key genes involved in biofilm formation such as *lasR*, *ladS*, *pqsA*, *pqsD*, *rhlI* and *retS* (Table S1). Interestingly, MS6000, MS6002 and MS6005, the isolates showing the most robust biofilm production, presented a premature stop codon in the gen *pelD* generated by a nucleotide substitution at position 731 (CAG by TAG), thus originating a truncated peptide at residue Q247. PelD is a cyclic diguanylate effector considered essential for biofilm production [83]. This observation led us to believe that alternative mechanism for biofilm production should exist in *P. aeruginosa*, as other plausible explanations are difficult to suggest from these experiments. Despite the large amount of information existing on biofilm production in *P. aeruginosa*, there are still many aspects to decipher as it is a multifarious mechanism, which depends not only on bacterial specific gene features but also on physiological and environmental conditions. In our case, more extensive experiments must be conducted to determine the actual contribution of those mutations to the differences observed in biofilm production in the CF *P. aeruginosa* isolates.

### 2.4. Virulence in Galleria mellonella

Virulence of the CF *P. aeruginosa* isolates was evaluated in the greater wax moth *G. mellonella*. Several studies have employed *G. mellonella* as a useful model host for investigating virulence traits of different human microbial pathogens [84].

**Figure 3:**
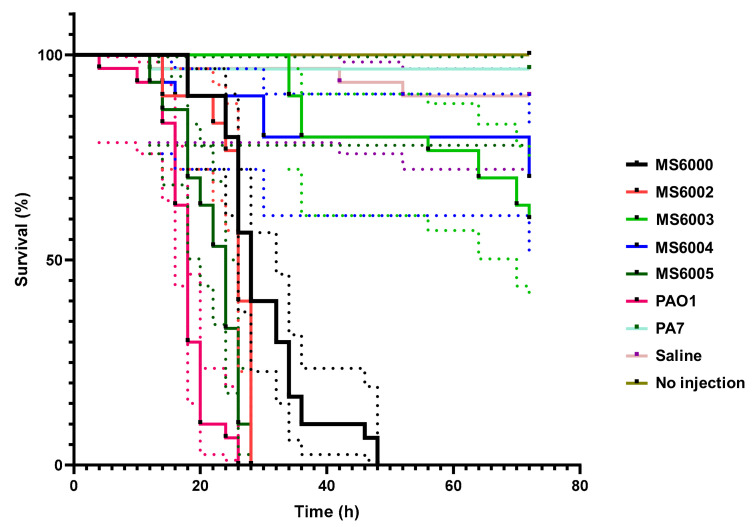
Kaplan–Meier survival curves of *G. mellonella* larvae after injection with 10 µL of 1 × 10^3^ CFUs of CF *P. aeruginosa* isolates of ST3449. Dead larvae were monitored every 2 h after inoculation up to 72 h. Uninoculated larvae and those inoculated with saline solution (0.85% NaCl), and *P. aeruginosa* strains PAO1 and PA7 were employed as control groups. The data shown represent three independent experiments with 95% confidence intervals, represented as dotted lines The isolates MS6002 and MS6005 showed high virulence capacity (killed 100% of the larvae at 28 h, similar to PAO1 that killed 100% of the larvae at 26 h) compared to that of MS6000 (killed 100% of the larvae at 48 h). Isolates MS6003 and MS6004 demonstrated very low virulence capacity.

*P. aeruginosa* PAO1 and PA7 were employed as high and low virulence control strains, respectively. The survival of isolates was monitored for 72 h after infection in order to determine their pathogenic potential. We found significant differences in larvae mortality among different groups (*p* < 0.05) (Figure 3). The most virulent isolates were MS6005, MS6002 and MS6000, showing median survival times of 24, 26 and 28 h, respectively and for control strain PAO1 18 h. In contrast, isolates MS6003 and MS6004 presented a very low virulence capacity, as at 72 h post inoculation more than 50% of larvae in both groups remained still alive.

Even when all our isolates presented similar number of virulence factors, the differences found in virulence patterns in *G. mellonella* reflects that virulence may be subjected to transcriptional regulation, depending on each isolate genetic background. Production of pigments are also associated with virulence in *P. aeruginosa*. As mentioned before our isolates displayed also differences in pigmentation, but we could not directly associate this phenotypic feature with larvae mortality observed in each group. Therefore, we corroborate that differences observed in the virulence patterns of the five isolates is not restricted to a certain number of virulence genes but it is a result of combinatorial mechanisms, which has been stated previously [85].

## 3. Materials and Methods

### 3.1. Bacterial Isolates and Antimicrobial Susceptibility Testing

Five consecutive *P. aeruginosa* isolates were collected during a month from respiratory samples (three from sputum and two from tracheal aspirate) from a cystic fibrosis patient with end-stage respiratory failure who died before lung transplantation.

The isolates were routinely grown on blood and/or Mueller–Hinton agar plates at 37 °C. Identification was performed by matrix-assisted laser desorption/ionization-time of flight mass spectrometry (MALDI-TOF/Vitek-MS with SARAMIS MS-IVD v2) [86]. Phenotypic traits such as colony morphology, hemolysis and pigment production were also examined.

The minimum inhibitory concentrations (MICs) of aztreonam, cefepime, ceftazidime, piperacillin, piperacillin-tazobactam, imipenem, meropenem, doripenem, amikacin, gentamicin, tobramycin, ciprofloxacin, levofloxacin and colistin were determined by broth microdilution according to CLSI guidelines, except for fosfomycin that was performed by agar dilution and the combinations of ceftolozane-tazobactam and ceftazidime-avibactam that were determined by Etest according to the manufacturer’s recommendations (bioMérieux, Marcy-I’Etoile, France). All antibiotics were purchased from Sigma-Aldrich. MIC breakpoints for all agents were those established by EUCAST 2021 v_11.0 (http://www.eucast.org/clinical_breakpoints/ (accessed on January 2021), except for fosfomycin that was done using an ECOFF (epidemiological cutoff) value of ≤128 µ/mL, and gentamicin for which CLSI breakpoints were applied. *Escherichia coli* ATCC 25,922 and *P. aeruginosa* ATCC 27,853 were employed as quality control strains. Susceptibility categories of multidrug resistant (MDR) and extensively drug resistance (XDR) were assigned to the strains according to the expert consensus recommendations previously reported [87].

### 3.2. DNA Extraction and Sequencing

Total DNA from the *P. aeruginosa* isolates was purified with the DNeasy Blood and Tissue Kit (Qiagen) and sequenced on a MiSeq device using reagents kit v3 for 2 × 300 paired-end libraries (Illumina) as previously described [88].

### 3.3. Bioinformatics Analysis

Raw reads from the sequencing platform were directly analyzed using the in-house bioinformatics pipeline TORMES^®^ [13]. *P. aeruginosa* PAO1 was used as a reference strain. The options used in this study included quality control and filtering of the reads by using Trimmomatic [89], Prinseq [90] and Kraken [91]. Genome assembly was performed with SPAdes [92] and Quast [93] and genome annotation with Prokka [94]. Multilocus sequence typing was performed using an open source tool (MLST, T. Seemann, https://github.com/tseemann/mlst (accessed on September 2020). Search of antibiotic resistance genes and plasmid replicons was done using BLAST [95] and ABRicate (https://github.com/tseemann/abricate (accessed on September 2020)) against ResFinder [19] and PlasmidFinder [96] databases, respectively. Search for single nucleotide polymorphisms (SNPs), insertion and deletions (indels) of nucleotides was performed with breseq [20]. Pangenome was created with Roary [97] and FastTree [98]. In silico serotyping was performed using the *P. aeruginosa* serotyper (PAst) program [15]. Plotting was carried out in R environment [99] by using ggplot2 [100], RColorBrewer [101] and reshape2 [102] packages. Blast Ring Image Generator (BRIG) [14] was used to show a genome wide visualization.

### 3.4. Biofilm Formation Assay

Biofilm formation was evaluated by means of the crystal violet staining assay as described before [103], with slight modifications. Briefly, *P. aeruginosa* overnight cultures were adjusted to an optical density at 600 nm (O.D_600_) of 0.2 in MHB. Biofilms were developed in 24 well plates (Nunc, Thermo Fisher Scientific). Subsequently, 100 µL of each bacterial suspension were placed in each well containing 900 μL of MHB and statically incubated at 37 °C for 24 h. Planktonic cells were removed and biofilms were washed three times with distilled water and air-dried for 20 min. Biofilms were stained with 1.5 mL/well of 0.7% crystal violet (wt/vol) solution (Sigma-Aldrich) for 12 min. The amount of dye (proportional to the density of adherent cells) was determined at 590 nm using a plate reader (Infinite^®^ 200 PRO, Tecan). Results were corrected for background staining by subtracting the value for crystal violet bound to uninoculated MHB control wells. The OD590/OD600 ratio was determined in order to normalize the amount of biofilm produced with respect to the total cell content in each sample to avoid variations due to differences in bacterial growth under different experimental conditions. The biofilm assay was performed six independent times. For the interpretation of biofilm results isolates were classified as non-biofilm producer (OD ≤ 0.05), weak-biofilm producer (OD > 0.05–0.1), moderate-biofilm producer (OD > 0.1–0.3) and strong-biofilm producer (OD > 0.3).

### 3.5. Confocal Laser Scanning Microscopy (CLSM)

Biofilm architecture was investigated by CLSM. Biofilms were developed in 4-well µ-slides (Ibidi, Martinsried, Germany) as previously described [104], but with few modifications. In brief, slides containing 500 µL of bacterial cultures (adjusted to an optical density OD_600_ = 0.2 in MHB), were placed inclined (45°) into an incubator at 37 °C in order to form a liquid–air interface. Planktonic cells were removed after 24 h, by rinsing three times with saline solution (0.85% NaCl), and then biofilms were stained with the FilmTracer^TM^ SYPRO Ruby Biofilm Matrix Stain (ThermoFisher Scientific) and 4′,6-diamidino-2-phenylindole (DAPI, Sigma-Aldrich) to reveal the biofilm protein components and nucleic acids, respectively. Both staining procedures were performed according to the manufacturer’s instructions. The stained biofilms were visualized under a Nikon A1R confocal laser scanning microscope (Nikon Instruments Inc., Melville, NY, USA). Six surfaces of six independent biological replicates were observed in each CLSM experiment, and representative images were selected. The excitation wavelengths were 405 nm and emission filters of 425–475 nm and 570–620 nm were used for DAPI and SYPRO, respectively. Measurements of biofilms 3D images were performed with the NIS-Element AR NiKon instrument software, version 3.2 (Nikon Instruments Inc., Melville, NY, USA).

### 3.6. Galleria mellonella Killing Assays

To assess the virulence of the *P. aeruginosa* isolates in vivo, the greater wax moth *G. mellonella* was used as a model of infection. Larvae of *G. mellonella* were obtained from Animal Center Valencia S.L (Valencia, Spain), stored in the dark on wood chips at 8–10 °C and used within 10 days of receipt. Larvae were selected to be 20–25 mm in length, presenting a healthy creamy color without speckles or grey markings.

Previous to inoculation, bacteria were grown overnight in MHB at 37 °C and the bacterial cultures were normalized using optical density (OD_600_) and the colony forming units (CFU/mL) were confirmed by the viable count assay. Bacterial inocula were then adjusted to 1 × 10^3^ CFUs with saline solution (0.85% NaCl) and 10 μL were injected into the hemocoel via the left proleg of each larva using a BD Micro-Fine sterile insulin syringe (0.33 mm (29G) × 12.7 mm, 500 µL capacity) (Becton Dickinson, Franklin Lakes, NJ, USA). The syringes were changed between treatments with different bacterial inocula. Two negative control groups were included; one group of 10 larvae were injected with 10 μL of saline solution in order to measure any lethal effect associated to the injection process and a second group of not injected larvae were used to measure the effects of the incubation procedure on larvae mortality. Two *P. aeruginosa* strains PAO1 [105] and PA7 [106] were used as controls for fast and slow killing assays, respectively. Three biological replicates of 10 larvae were used for each experimental condition. Larvae were then incubated in petri dishes at 37 °C in the dark under aerobic conditions and survival was recorded at 2 h intervals for 72 h. Larvae were considered dead when they displayed complete melanization and/or no response was observed following touch.

### 3.7. Statistical Analyses

All statistical analyses were carried out using GraphPad Prism version 9.0.2 (GraphPad Software, San Diego, CA, USA). All experiments were performed at least six times and the results were shown as means ± standard deviation. Differences between mean values were tested for significance by performing one-way ANOVA analysis followed by Tukey’s multiple-comparison test, when appropriate. Survival curves were plotted using the Kaplan–Meier method, and differences in survival were calculated using the log-rank (Mantel–Cox) test for multiple comparisons. In all cases differences were considered statistically significant at *p* < 0.05.

### 3.8. Nucleotide Accession Numbers

The whole-genome sequencing reads and annotated assembly of the five *P. aeruginosa* isolates of ST3449 are available under the BioProject ID PRJNA695127.

## 4. Conclusions

This work is relevant as it identified and characterized five consecutive clonally related multidrug-resistant *P. aeruginosa* isolates (four of them XDR) that belong to a novel sequence type ST3449 and serotype O6, from a CF patient with end-stage pulmonary disease, who died before lung transplantation.

Our principal aim was the whole-genome sequence analysis of these isolates showing diverse phenotypic features such as changes in pigmentation, colony morphology, biofilm production and virulence capacity. This study demonstrates that the isolates analyzed here are probably the ones that presented a better fitness to subsist in the CF lung environment at the end-stage patient’s disease. Other isolates with less capacity of adaptation or reduced fitness might have coexisted at the same spatial time, but we could not isolate those ones from early-phase (primo-colonization) or mid-term colonization. Our isolates were collected during a month, and the patient had died already when the isolates were examined. Analysis of earlier isolates would have allowed us to compare the evolution of this novel *P. aeruginosa* clone ST3449 or the possible presence of other clones coexisting within a defined period of time.

Our observations corroborate previous studies regarding the coexistence of clonally related *P. aeruginosa* strains showing different phenotypic and genotypic characteristics within the lung of CF patients, which is a reflect of the enormous capacity of adaptation of this pathogen to the hostile environment in the CF lung. Due to the WGS we could describe new mutation in several genes related with antimicrobial resistance, virulence and other cell functions, which reflects the high pressure *P. aeruginosa* experiences in the lung of CF patients to generate new allelic variants of these genes in order to persist and survive in such harsh environment. On the other hand we could observe that some mutations in antimicrobial resistance related genes are possible a distinctive feature of *P. aeruginosa* isolates from CF as they repeatedly occur in these isolates.

Despite not performing any experiment to demonstrate mutation frequencies, all our isolates are very likely hypermutable, as a number of mutations were described in some genes known to be responsible for the hypermutator phenotype.

We believe that our results provide some new valuable information about the evolution and dynamics of antimicrobial resistance and virulence mechanisms seen through the repertoire of mutational events accumulated by some *P. aeruginosa* clones as is the case of this novel ST3449, described here for the first time in a CF patient. We consider it is important to continue characterizing and monitoring this new clone due to the potential risk it may represent for human health.

## Figures and Tables

**Table 1:** Phenotypic characteristics of the CF *P. aeruginosa* isolates.

Isolate *	Source	Colony Morphology	Pigmentation	Hemolysis
MS6000	Sputum	Small, mucoid	Brown	+
MS6002	Sputum	Large, irregular	Blue-green opaque	+
MS6003	Sputum	Small, circular	Green brilliant	+
MS6004	Tracheal aspirate	Small, wrinkled	Green opaque	+
MS6005	Tracheal aspirate	Large, irregular	Brown	+

* Isolate numbering was assigned according to isolation date.

**Table 2:** Antimicrobial susceptibility of *P. aeruginosa* ST3449 isolates.

Isolate	CMI (µg/mL)																	Resistance Phenotype
	AZT	FEP	CAZ	C/A *	C/T *	PIP	TZP	IMP	MER	DOR	GEN **	TOB	AK	CIP	LEV	COL	FOS ***	
MS6000	64 ^(R)^	16 ^(R)^	64 ^(R)^	4 ^(S)^	0.5 ^(S)^	256 ^(R)^	256 ^(R)^	1 ^(I)^	0.25 ^(S)^	0.25 ^(S)^	8 ^(I)^	4 ^(R)^	16 ^(S)^	0.5 ^(I)^	2 ^(R)^	2 ^(S)^	256 ^(R)^	XDR
MS6002	64 ^(R)^	8 ^(I)^	16 ^(R)^	2 ^(S)^	0.38 ^(S)^	64 ^(R)^	128 ^(R)^	32 ^(R)^	16 ^(R)^	8 ^(I)^	8 ^(I)^	0.5 ^(S)^	8 ^(S)^	1 ^(R)^	4 ^(R)^	8 ^(R)^	512 ^(R)^	XDR
MS6003	8 ^(I)^	16 ^(R)^	16 ^(R)^	1 ^(S)^	0.75 ^(S)^	128 ^(R)^	4 ^(I)^	8 ^(R)^	1 ^(S)^	2 ^(S)^	16 ^(R)^	1 ^(S)^	32 ^(R)^	>32 ^(R)^	>32 ^(R)^	4 ^(R)^	128 ^(S)^	XDR
MS6004	0.25 ^(I)^	0.5 ^(I)^	1 ^(I)^	0.125 ^(S)^	0.125 ^(S)^	0.5 ^(I)^	1 ^(I)^	1 ^(I)^	0.06 ^(S)^	0.125 ^(S)^	4 ^(S)^	1 ^(S)^	8 ^(S)^	2 ^(R)^	4 ^(R)^	2 ^(S)^	64 ^(S)^	MDR
MS6005	>128 ^(R)^	128 ^(R)^	128 ^(R)^	3 ^(S)^	0.25 ^(S)^	>256 ^(R)^	256 ^(R)^	4 ^(I)^	2 ^(S)^	2 ^(S)^	8 ^(I)^	4 ^(R)^	16 ^(S)^	4 ^(R)^	16 ^(R)^	2 ^(S)^	512 ^(R)^	XDR

AZT, aztreonam; FEP, cefepime; CAZ, ceftazidime; C/A, ceftazidime/avibactam; C/T, ceftolozane/tazobactam; PIP, piperacillin; TZP, piperacillin/tazobactam; IPM, imipenem; MEM, meropenem; DOR, doripenem; GEN, gentamicin, TOB, tobramycin; AK, amikacin; CIP, ciprofloxacin; LEV, levofloxacin; COL, colistin; FOS, fosfomycin.; * MICs of these antibiotics were determined by Etest; ** MIC interpretations were according to CLSI breakpoints; *** ECOFF used by EUCAST was applied for fosfomycin MIC interpretations: susceptible, ≤ 128 µg/mL; and non-susceptible or resistant, > 128 µg/mL; ^(S)^, susceptible; ^(I)^, intermediate; ^(R)^, resistant. MDR, multidrug-resistant; XDR, extensively drug-resistant.

**Table 3:** Genome assembly summary of the CF *P. aeruginosa* isolates.

Sample	Reads	Average Read Length (bp)	Contigs	Genome Length (bp)	Average Contig Length (bp)	N50 (bp)	ORF	GC Content	Depth	Species	Percent ID
MS6000	629064	278	119	6,326,892	576,053	262,138	5815	66.37	27X	*P. aeruginosa*	99.29
MS6002	876966	279	109	6,324,360	926,907	375,329	5830	66.38	38X	*P. aeruginosa*	99.35
MS6003	877052	277	123	6,339,440	920,338	351,508	5836	66.35	38X	*P. aeruginosa*	99.28
MS6004	915080	278	105	6,451,152	777,263	281,904	5953	66.37	39X	*P. aeruginosa*	98.61
MS6005	773800	278	122	6,334,587	777,177	351,513	5826	66.35	33X	*P. aeruginosa*	99.30
Average	814392	278	116	6,355,286	795,548	324,478	5852	66.36	35X	-	99.37

**Table 4:** Alterations in proteins/genes related with antimicrobial resistance found in the CF *P. aeruginosa* isolates of ST3449.

Protein/Gene	MS6000	MS6002	MS6003	MS6004	MS6005
AmpC	T105A	T105A	T105A	T105A	T105A
AmpR	**+T at nt 860, stop TGA at 874**	**+T at nt 860, stop TGA at 874**	**+T at nt 860, stop TGA at 874**	**+T at nt 860, stop TGA at 874**	**+T at nt 860 stop TGA at 874**
AmpD	G148A, D183Y	G148A, D183Y	D183Y, +G at 443	G148A, D183Y	G148A, D183Y
AmpDh2	wt	wt	wt	wt	wt
AmpDh3	A219T	A219T	A219T	A219T	A219T
*mpl*	**Del. 14 bp (1120–1133)**	**Del. 14 bp (1120–1133)**	wt	G133D	**Del. 14 bp (1120–1133)**
PBP1a (*ponA*)	**D329G, +CCG at nt 1845**	**D329G, +CCG at nt 1845**	**D329G, +CCG at nt 1845**	**D329G, +CCG at nt 1845**	**D329G, +CCG at nt 1845**
PBP1b (*mrcB*)	L353Q	L353Q	L353Q	L353Q	L353Q
PBP3a (*pbpC*)	Not present	Not present	Not present	A104P	Not present
PBP7 (*pbpG*)	S250N	S250N	S250N	S250N	S250N
MexA	wt	wt	wt		wt
MexB	**L672R**	**L672R**	**L684P**	**L684P**	**L672R**
OprM	wt	wt	wt	wt	wt
MexR	wt	wt	wt	wt	wt
NalC	G71E, S209R	G71E, S209R	G71E, S209R	G71E, S209R	G71E, S209R
NalD	wt	wt	wt	wt	wt
OprD	D43N, S57E, S59R, E202Q, I210A, E230K, S240T, N262T, A267S, A281G, K296Q, Q301E, R310G, V359L, M372V, S373D, D374S, N375S, N376S, V377S, Del.G378, Del.Y379, K380Y, N381A, Y382G, G383L	Stop at nt 18 (TGG→TGA) W6 *	D43N, S57E, S59R, E202Q, I210A, E230K, S240T, N262T, A267S, A281G, K296Q, Q301E, R310G, V359L, M372V, S373D, D374S, N375S, N376S, V377S, Del.G378, Del.Y379, K380Y, N381A, Y382G, G383L	D43N, S57E, S59R, E202Q, I210A, E230K, S240T, N262T, A267S, A281G, K296Q, Q301E, R310G, V359L, M372V, S373D, D374S, N375S, N376S, V377S, Del.G378, Del.Y379, K380Y, N381A, Y382G, G383L	D43N, S57E, S59R, E202Q, I210A, E230K, S240T, N262T, A267S, A281G, K296Q, Q301E, R310G, V359L, M372V, S373D, D374S, N375S, N376S, V377S, Del.G378, Del.Y379, K380Y, N381A, Y382G, G383L
MexX	K329Q, L331V, W358R	K329Q, L331V, W358R	K329Q, L331V, W358R	K329Q, L331V, W358R	K329Q, L331V, W358R
MexY	T543A, **A992T**	T543A, **A992T**	T543A	T543A	T543A, **A992T**
MexZ	wt	wt	wt	wt	wt
AmgS	wt	wt	wt	wt	wt
Fmt	I181V, E277K	I181V, E277K	I181V, E277K	I181V, E277K	I181V, E277K
FolD	T189A	T189A	T189A	T189A	T189A
FusA2	S176A, G695A	S176A, G695A	S176A, G695A	S176A, G695A	S176A, G695A
NuoG	T484A	T484A	T484A	T484A	T484A
MexC	S330A	S330A	S330A	S330A	S330A
MexD	E257Q, S845A	E257Q, S845A	E257Q, S845A	E257Q, S845A	E257Q, S845A
OprJ	M69V	M69V	M69V	M69V	M69V
NfxB	wt	wt	wt	wt	wt
MexT	Del. GCCGGCCA (240–247), F172I	Del. GCCGGCCA (240–247), F172I	Del. GCCGGCCA (240–247), F172I	Del. GCCGGCCA (240–247), F172I	Del. GCCGGCCA (240–247), F172I
MexE	wt	wt	wt	wt	wt
MexF	wt	wt	wt	wt	wt
OprN	wt	wt	wt	wt	wt
MexS	D249N	D249N	D249N	D249N	D249N
GyrA	D87N	D87N	T83I	T83I	D87N
GyrB	S618L	S618L	wt	wt	S618L
ParC	V646L	V646L	E91K, V646L	E91K, V646L	E91K, V646L
ParE	G285S	G285S	G285S	G285S	G285S
CrpP	K4R, G7D	K4R, G7D	K4R, G7D	K4R, G7D	K4R, G7D
PmrA	L71R	L71R	L71R	L71R	L71R
PmrB	Y345H, V386M	Y345H, V386M	Y345H, V386M	Y345H, V386M	Y345H, V386M
ParR	L153R, S170N	L153R, S170N	L153R, S170N	L153R, S170N	L153R, S170N
ParS	H398R	H398R	Stop at nt 459 (TGG→TGA) W153 *	H398R	H398R
ArnB	V302A, E376D	V302A, E376D	V302A, E376D	V302A, E376D	V302A, E376D
ArnT	I509V	I509V	I509V	I509V	I509V
ArnF	T106I	T106I	T106I	T106I	T106I
PA3559 (ugd)	V101A	V101A	V101A	V101A	V101A
OprP	A98V	A98V	A98V	A98V	A98V

The plus (+) sign indicates addition of a base at a certain position in the DNA sequence of the gene. In bold are represented those mutations/polymorphisms that to the best of our knowledge are described here for the first time. Del. (deletion). (*) an asterisk indicates that the protein is truncated from the amino acid residue due to the presence of a premature stop codon at a certain position of the sequence of the gene.

## Data Availability

The data presented in this study are in Supplementary Tables S1 and S2. Genome sequences information is available in https://www.ncbi.nlm.nih.gov/bioproject/ (accessed on 26 January 2021) under the BioProject ID PRJNA695127.

## Supplementary Materials

The following are available online at https://www.mdpi.com/article/10.3390/antibiotics10050491/s1, Table S1: Mutations found in the WGS analysis of the *P. aeruginosa* isolates from ST3449. Table S2: Virulence genes of *P. aeruginosa* isolates from ST3449. Figure S1: Correlation between biofilm formation and quantification methods.
