# Supplementary material for: New Sequence Type ST3449 in Multidrug-Resistant Pseudomonas aeruginosa Isolates from a Cystic Fibrosis Patient
Source: Antibiotics (Basel). 2021 Apr 23;10(5):491. doi: 10.3390/antibiotics10050491 (PMC8146123; doi:10.3390/antibiotics10050491)
Supplement: Supplementary file 1 [file antibiotics-10-00491-s001.zip › Figure S1.pdf]

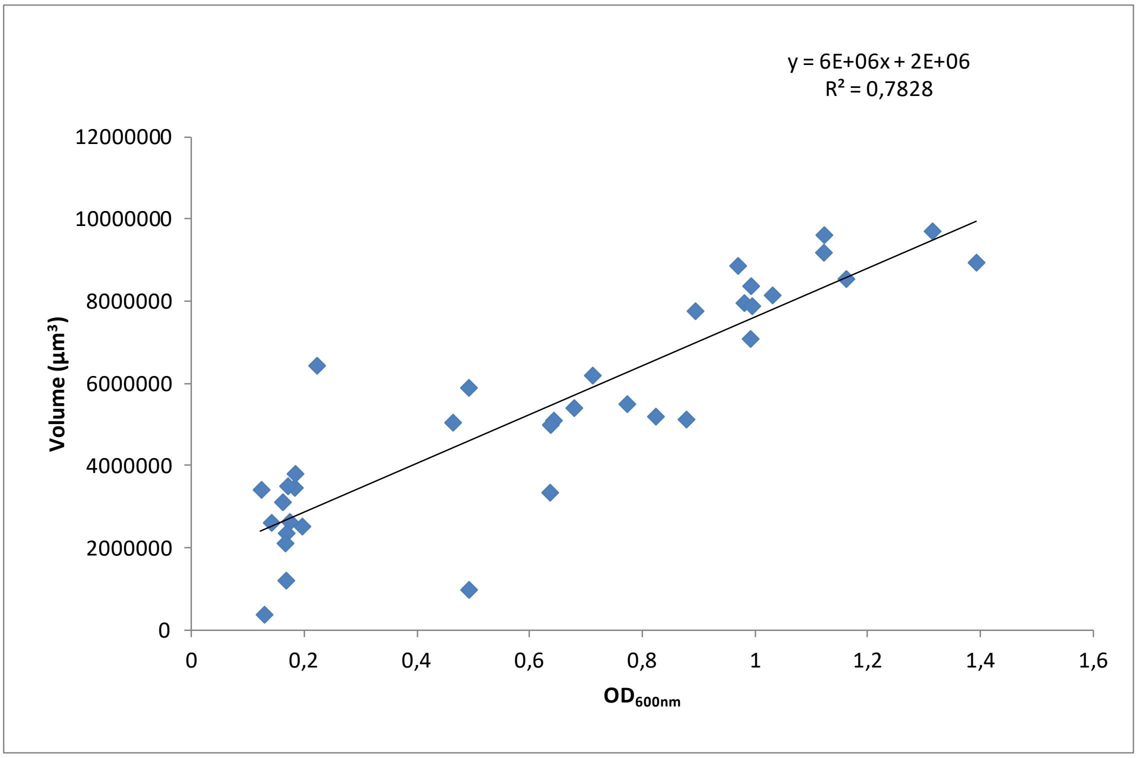

Figure S1. Correlation between the biofilm formation and quantification methods. A substantial correlation (R^2=0.7828) between the crystal violet and Film tracer SYPRO Ruby biofilm matrix staining methods was observed.
